# Prevention of Overweight in Infancy (POI.nz) study: a randomised controlled trial of sleep, food and activity interventions for preventing overweight from birth

**DOI:** 10.1186/1471-2458-11-942

**Published:** 2011-12-19

**Authors:** Barry J Taylor, Anne-Louise M Heath, Barbara C Galland, Andrew R Gray, Julie A Lawrence, Rachel M Sayers, Kelly Dale, Kirsten J Coppell, Rachael W Taylor

**Affiliations:** 1 Department of Women's and Children's Health, University of Otago, PO Box 913, Dunedin 9016, New Zealand; 2 Department of Human Nutrition, University of Otago, PO Box 913, Dunedin 9016, New Zealand; 3 Department of Preventive and Social Medicine, University of Otago, PO Box 913, Dunedin 9016, New Zealand; 4 Edgar National Centre for Diabetes and Obesity Research, University of Otago, PO Box 913, Dunedin 9016, New Zealand

## Abstract

**Background:**

Rapid weight gain during the first three years of life predicts child and adult obesity, and also later cardiovascular and other morbidities. Cross-sectional studies suggest that infant diet, activity and sleep are linked to excessive weight gain. As intervention for overweight children is difficult, the aim of the Prevention of Overweight in Infancy (POI.nz) study is to evaluate two primary prevention strategies during late pregnancy and early childhood that could be delivered separately or together as part of normal health care.

**Methods/Design:**

This four-arm randomised controlled trial is being conducted with 800 families recruited at booking in the only maternity unit in the city of Dunedin, New Zealand. Mothers are randomised during pregnancy to either a usual care group (7 core contacts with a provider of government funded "Well Child" care over 2 years) or to one of three intervention groups given education and support in addition to "Well Child" care: the Food, Activity and Breastfeeding group which receives 8 extra parent contacts over the first 2 years of life; the Sleep group which receives at least 3 extra parent contacts over the first 6 months of life with a focus on prevention of sleep problems and then active intervention if there is a sleep problem from 6 months to 2 years; or the Combination group which receives all extra contacts. The main outcome measures are conditional weight velocity (0-6, 6-12, 12-24 months) and body mass index z-score at 24 months, with secondary outcomes including sleep and physical activity (parent report, accelerometry), duration of breastfeeding, timing of introduction of solids, diet quality, and measures of family function and wellbeing (parental depression, child mindedness, discipline practices, family quality of life and health care use). This study will contribute to a prospective meta-analysis of early life obesity prevention studies in Australasia.

**Discussion:**

Infancy is likely to be the most effective time to establish patterns of behaviour around food, activity and sleep that promote healthy child and adult weight. The POI.nz study will determine the extent to which sleep, food and activity interventions in infancy prevent the development of overweight.

**Trial Registration:**

Clinical Trials NCT00892983

Prospective meta-analysis registered on PROSPERO CRD420111188. Available from http://www.crd.york.ac.uk/PROSPERO

## Background

Obesity is one of the greatest threats to health in the 21st century in many countries, including New Zealand. New Zealand is one of the five worst OECD countries for both child and adult overweight status [1], with almost 10% of children aged 5-14 years currently considered obese and a further 20% identified as overweight [2]. Many parents do not recognise that their children are overweight [3,4], and even if they do, long-term outcomes from interventions once children or adults are overweight are not impressive [5,6]. In response to convincing evidence that early growth sets the pattern for future growth and predicts both childhood and adult obesity, as well as later cardiovascular morbidity and mortality [7-9], there has been an increasing focus on preventive interventions during infancy.

In designing interventions for the primary prevention of excessive weight gain in infancy, it appears logical to begin with the most obvious determinants of energy balance--energy intake (breastfeeding, complementary feeding, and infant diet) and energy expenditure (activity and sedentary behaviours). However, over the last 10 years, increasing evidence has also linked sleep problems, and in particular short sleep duration, with increased risk of excessive weight gain [10]. An intervention that seeks to alter infant food, activity and sleep needs to effectively modify parental practices in these areas in order to positively influence infant behaviour. The following sections outline the justification for our approach.

### Breastfeeding and introduction of complementary foods

Despite continued controversy, a recent review of systematic reviews of observational studies supports an association between no, or short, duration of breastfeeding and an increased risk of overweight and obesity to 5 years of age [11]. Although the effect appears to be small--each additional month of breastfeeding may be associated with a 4% decrease in the odds of overweight [12]--it does not appear to be explained by publication bias or confounding [13]. Breastfeeding may be particularly protective against obesity if the breastfeeding is exclusive [14], or the mother is overweight [15]. However, limited intervention studies have been performed and it is not possible to conduct a trial in which infants are randomised to receive or not receive breast milk. The PROBIT study was able to randomise maternity hospitals in Belarus to either a breastfeeding promotion based on the WHO-UNICEF Baby-Friendly Hospital Initiative, or standard practices and policies in place at the time of the study. Although the intervention group increased their duration of exclusive breastfeeding this had no effect on body mass index (BMI) at 6.5 years of age [16]. However, caution must be exercised when applying these results to countries such as New Zealand, where obesity rates, and therefore the potential for prevention, are considerably higher.

It has been suggested that later introduction of complementary foods may be associated with lower percentage body fat at age 7 years [17] and decreased risk of adult overweight [18]. However, this is controversial [19], with a number of studies finding no immediate effect on growth during infancy including two small randomised trials to 6 and 12 months of age [20,21]. More recently, a longitudinal study following Australian infants from before birth to 10 years of age found that delaying the introduction of solids from 20 to 24 weeks of age was associated with a 10% lower odds of overweight or obesity at 10 years from multivariate modelling [22]. The authors propose potential mechanisms, including epigenetic modification of metabolic programming and effects of excess protein intake on age of adiposity rebound that may account for a delayed effect on obesity, even in the absence of an immediate effect on body weight.

### Food and eating

Early studies clearly demonstrated that children are born with an ability to self-regulate intake to match physiological needs [23]. However, the rising prevalence of overweight in toddlers and preschool-aged children and the current mismatch between energy intake and requirements [24] would suggest that this ability is either lost or ignored from fairly early in life. The potential role that parents play in encouraging appropriate eating is thus of considerable interest. In feeding terms, responsive parenting refers to a parent promoting a pleasant feeding environment and responding to hunger and satiety cues in their offspring. By contrast, unresponsive parenting involves over-control from the parent such as pressuring the child to eat or overtly restricting foods, too little control of the child's eating (uninvolved feeding), or too much control by the child (indulgent feeding) [25]. The majority of studies investigating responsive parenting in young children demonstrate significant inverse relationships with body weight [26]. Ideally parents play a role in helping children develop appropriate food preferences by offering a range of healthy foods, on a number of occasions, and in a relaxed environment [27].

Factors other than parenting style have also been implicated in excessive weight gain during the early years, including family meals, portion size, and consumption of sweetened beverages. Family meals are associated with healthy eating patterns [28] and reduced obesity [29] in children and adolescents. However, the relative effects may differ across ethnic and socioeconomic groups [30] and any positive effects of shared mealtimes may be mitigated by having television on at the same time [28]. Although the precise mechanisms remain to be elucidated, eating together allows modelling of eating practices, is predictive of family connectedness and presumably facilitates communication.

Portion size has also been implicated in the development of obesity [31] and experimental studies consistently demonstrate that offering larger portions of energy-dense foods results in a greater intake of food, at least in the short-term [32,33]. In contrast, providing larger portions of low energy-dense foods (fruit and vegetables) increases the overall intake of these foods, without increasing total energy intake for the meal [34]. However, educating parents about appropriate portion sizes for children is complicated by a lack of awareness or indeed concern about age-appropriate portion sizes for children [35]. Furthermore, although earlier work suggested that preschool-aged children may be relatively immune to the obesogenic effect of increased portion size [36], more recent studies highlight that energy dysregulation may occur as early as 12 months of age [37,38]. Training parents to recognise and respond to hunger and satiety cues in their infants in an effort to encourage appropriate food consumption independently of portion size is therefore of considerable interest.

The relative contribution of sweetened beverages to the development of obesity is controversial [39,40]. There is no doubt that intakes of sweetened beverages have increased substantially over time in all age groups, at least in the US where the majority of infants aged 6-24 months consume sweetened beverages, predominantly fruit juice or fruit drinks (6 ounces/day) with smaller intakes (1.5 ounces/day) of other sweet drinks [41], at a time when obesity rates have risen. Certainly there appear to be links between consumption of sweet beverages and body weight in older preschool children [42].

### Physical activity and sedentary behaviours

Engaging in more sedentary activities (including television viewing) and less outside play have also been linked to the development of obesity with some evidence suggesting that less active pre-school children remain less active than their peers throughout childhood [43]. Although it is commonly believed that young children are very active, studies utilising objective measures of physical activity (accelerometry) demonstrate that even preschool aged children spend a large portion of their day in sedentary activities, as do older children and adolescents [44,45]. Expert groups currently recommend exposing infants to prone play or "tummy time" to help facilitate motor milestone development, and limiting the time spent restrained in car seats, strollers, prams, and play pens [46]. However many parents do not encourage prone play in their infants, because of either infant resistance, or misperceptions around positioning during sleep (prone not recommended) and awake (prone recommended) [47].

Despite recommendations from expert groups to discourage television viewing in children under two years of age [48], television viewing is common in this age group [49]. This applies to even the youngest infants, with 40% of 3 month olds viewing television, a proportion that increases with age so that 90% of 24 month old children are watching television regularly [50]. Exposure to television in the infant and toddler years has been associated with irregular sleep patterns [51] and poorer dietary habits [52], and may impair later language and cognitive development [53], and predispose children to obesity [54]. Interventions to reduce television viewing in the first few years of life show promising effects on the time spent watching television [55,56].

### Sleep

At birth, the sleep of term infants is considered polycyclic, i.e. there are multiple sleep periods and wake periods in a 24-hour day. On average, infants from birth to 2 months sleep approximately 15 h with short periods of waking [57]. Gradually infants consolidate their sleep into nocturnal sleep and daytime wake, coincident with maturation of sleep-wake regulatory systems within the brain circuitry. However, behavioural or physiological factors can intervene to upset sleep-wake regulatory systems, resulting in problematic sleep. Parenting is proposed to play a key role in sleep-wake regulatory systems [58]. Sleep problems within the normal (non-clinical) population fall into the categories of: 1) insomnia (difficulty getting to sleep); 2) poor sleep maintenance (difficulty staying asleep), and; 3) sleep fragmentation (frequent night waking). Sleep problems can result in a shorter than normal sleep duration measured over a 24-hour period (termed "short sleep duration").

Adequate sleep in infancy and childhood is important for physical and psychosocial growth and development. A number of cross-sectional and longitudinal studies have linked short sleep duration with the development of obesity [10,59,60]. Moreover, as obesity rates have increased over the last one or two decades, some longitudinal studies have reported a significant decline in sleep duration [61,62] possibly associated with modern lifestyle.

Sleep difficulties in early infancy are common (25-35% prevalence) and can be transient but those unable to achieve a sleep duration of 6 h by 5 months of age have a much greater risk of short sleep duration and sleep problems later in childhood [63,64]. Moreover, sleep problems often co-exist with feeding difficulties [65]. There are proven strategies for preventing and treating sleep problems in infancy [66], which may also decrease postnatal depression in mothers and improve family well-being [67]. However, their potential impact on growth and obesity is not known. In one study these sleep interventions did not appear to affect growth [68] but the effects of the sleep intervention on parent-reported sleep problems were not large, there were no objective measures of sleep, and growth outcomes were not part of a pre-planned analysis. Paul et al. [69], however, report a pilot study of a combination infant sleep intervention and weaning intervention that had a significant effect on weight for height centiles at 1 year of age with the mean weight for height centile in the combined intervention group being the 33^rd ^centile compared to the 50^th ^centile for the comparison group.

### Other factors

At least 9 genes have been associated with obesity in childhood [70], and at least one of these seems to influence appetite [71]. As genes and environment interact at many levels, it is important to assess the more common genetic loci increasing the risk for excessive weight gain in large intervention studies.

All parenting practices, including those around infant food, activity and sleep, are influenced by parenting style, a psychological construct representing standard strategies that parents use in their child rearing. Parenting style is characteristic of the parent, is not altered by the child, is stable over time and acts as the context that moderates the influences of specific parenting practices [72]. It is therefore important to investigate the relationship between parenting style and the success of any behaviour intervention. Managing children's behaviour is a fundamental part of parenting, and disciplinary practices within families that can have a lifelong effect on children's well-being [73]. Surprisingly few studies have explored the emergence of discipline practices in the first few years of life and those authors who have, have focused on physical discipline [74,75]. More recently, research has suggested that punishment of any type, and verbal aggression, may have the same adverse effects on well-being as physical discipline [76]. Several American studies have claimed that as many as a third of parents reported yelling at infants before they were nine months of age and twice this number between 19 and 35 months [77,78]. As education and support around the areas of feeding, activity and sleep are part of broader parental efforts to modify child behaviour, examining the potential effects (either positive or negative) on child discipline of any intervention that affects parenting behaviour is warranted.

### Aim

The aim of this study is to evaluate the effect on weight velocity and body mass index at 24 months of age of two early childhood obesity prevention interventions delivered to parents in late pregnancy and the first 2 years of their infant's life: anticipatory guidance, extra education and support to encourage (a) positive diet and physical activity behaviours, or (b) appropriate sleeping patterns, or (c) both interventions combined.

## Methods/Design

### Overall study design

**Figure 1:**
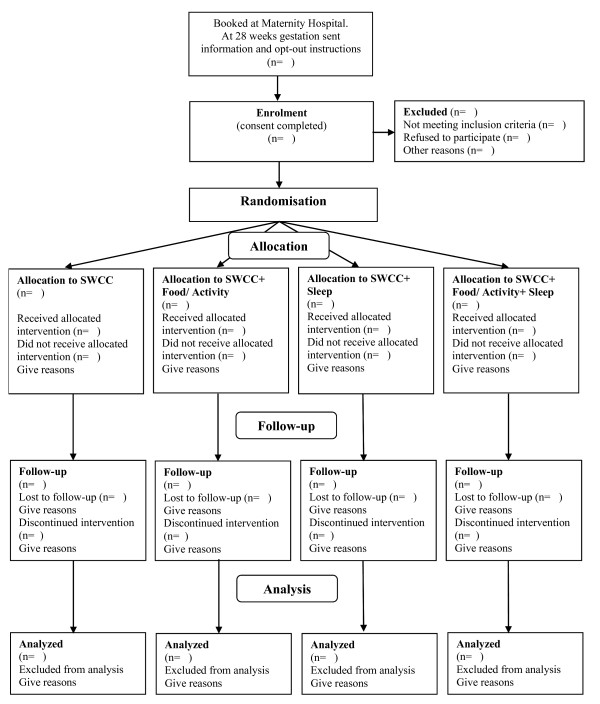
**Consort diagram for POI.nz study**. SWCC stands for Standard Well Child Care.

The study is a 4 arm randomised controlled trial (see Figure 1), consisting of a two-year intervention phase with planned follow-up for 5 years depending on additional funding. Ethical approval to conduct this study has been granted by the New Zealand Lower South Regional Ethics Committee (Project Key: LRS/08/12/063).

### Participants and recruitment

All pregnant women booked into the Queen Mary Maternity Unit, Dunedin Hospital (Dunedin, New Zealand), will receive a letter acknowledging their booking and providing initial information about the study. There is no other birthing facility in Dunedin, and the number of home births in Dunedin is < 3%. The Lead Maternity Carers (LMCs) who offer home births will give similar information to women requesting home births. At 28 weeks gestation the prospective participant will be sent a letter inviting participation with an opt-out option. Should the woman not opt-out, a research nurse will ring her within 2 weeks to establish eligibility, explain the study, answer any initial questions and, if appropriate, make a time for an individual meeting so that the woman can give her written informed consent to participate. Once eligibility is established and consent obtained, the woman will be randomised to one of the four study groups.

#### Inclusion criteria

Women will be eligible to participate if they are aged 16 years and over; are booked into the birthing unit at Queen Mary Maternity Unit, Dunedin Hospital, or we are notified by their home birth LMC, before 34 weeks gestation; are able to communicate in English or te reo Māori (Māori language); and are not planning to leave the local area prior to their child's second birthday.

#### Exclusion criteria

After birth, babies will be excluded if they are born before full term (36.5 weeks), or if a congenital abnormality or a physical or intellectual disability likely to affect feeding, physical activity or growth is identified.

### Sample size

A sample of 800 mothers (200 in each arm) will be required for the study at baseline, allowing for approximately 25% drop-out. The sample size calculation was based on detecting differences in the two main outcomes: weight velocity and body mass index (BMI) at two years with power set at 80% and two-sided significance (alpha) at 5% in all cases. A sample size of 103 per group is needed to detect a difference in the proportion of children having a weight velocity above the 75th centile of the World Health Organisation (WHO) "ideal growth" standards of 50 versus 30% in any two arms of the trial at 3 years. One hundred and forty-two participants per group would be required to detect a difference in BMI of 0.5 kg/m^2 ^using a mean (SD) of 16.8 (1.5) from an earlier study [79]. Our study is also sufficiently powered to detect differences in several secondary outcomes including a difference in 24-hour sleep duration of 0.5 h (23 participants per group assuming a SD of 0.6 h) and a difference in sleep problems of 35% versus 20% (151 per group).

### Registration and randomisation

A computerised random-number generator will be used to assign blocks of participants to the four arms. As socioeconomic status (SES) and parity may significantly affect the primary outcomes, stratified block allocation was used (SES low, medium or high (defined by the 2006 New Zealand Index of Deprivation [80]); parity 1 vs. > 1) with a block size of three used within each combination of strata. Allocation will be concealed and performed following application of the inclusion and exclusion criteria. The randomised group will be revealed to the participant by the research nurse on the opening of an opaque pre-sealed envelope.

### Study groups

#### Usual care group

Families in the Usual Care group (the comparison group) will receive the standard government funded "Well Child" care service which includes 7 core visits at 2-4 weeks, 6 weeks, 3, 5, 8-10 and 15 months, and 2 years of age respectively. These free home and clinic visits with a Well Child nurse assess growth and development, hearing, vision and wellness for all children in New Zealand (see http://www.health.govt.nz/our-work/life-stages/child-health/well-child-services/well-child-overview). The nurses offer support and guidance with growth and development assessments and information about breastfeeding, nutrition, parenting, safe sleep environments, smoking cessation, safety, immunisation, and family health issues.

#### Intervention groups

Families randomised to one of the three intervention groups will receive standard Well Child care *plus *additional support and education according to group allocation. The interventions will be delivered by specially trained research nurses, lactation consultants, and sleep specialists as appropriate via home visits (majority) and group sessions, as described below.

##### FAB group

Those in the Food, Activity and Breastfeeding (FAB) group will receive an additional 8 parent contacts for augmented education and support around breastfeeding, food and activity over the first 18 months. An anticipatory guidance group session will be held before birth (typically 34-35 weeks gestation) to discuss breastfeeding (benefits, challenges, developing a "breastfeeding plan") and outline the FAB programme. A registered lactation consultant will visit each mother in the first week she returns home from the maternity unit and when the infant is 4 months of age to provide specific support and education around breastfeeding (exclusive breastfeeding to 6 months of age, breastfeeding to 12 months of age), or formula feeding if necessary, and the introduction of solid foods (delaying to 5-6 months of age, re-offering new foods that have been rejected, beverages, family meals, hunger and satiety cues, "you provide, they decide"). The lactation consultant will also be available for additional support when requested by the participant up to 6 months postpartum. Group physical activity sessions will be run by early childhood exercise specialists at 3, 9 and 18 months. These sessions target the importance of awake prone time ("tummy time"), outdoor play, and family activity; and encourage limiting television viewing prior to two years of age. Food-based education sessions will be held at 7, 12 (individual) and 18 (group) months of age aiming to improve several aspects of food intake (fruit, vegetables, snack foods, treat foods, beverages, family meals, breakfast, portion sizes) as well as providing advice on appropriate parenting strategies via the use of a "you provide, they decide" model [81], with repeated offering of new foods that have been rejected.

##### Sleep group

Those in the Sleep group receive an additional 3 (minimum) parent contacts focussing on developing appropriate sleep habits from early in life. An anticipatory guidance group session will be held before birth (typically 34-35 weeks gestation) to outline normal sleep and what to expect in the first few months, as well as techniques to prevent the development of sleep problems--primarily detecting signs of tiredness early and placing baby to sleep awake but just before they go to sleep, maximising day-night differences, and avoiding bed-sharing. A research nurse then conducts home visits at 3 weeks, 4 months and 6 months, with sleep advice given as required. At the 3 week home visit, a sleep information booklet is given to participants and the information is re-enforced in one to one interaction with the research nurse. Parents who indicate that their child has problematic sleep at any time from 6 months to 2 years of age are offered a more intensive sleep intervention. If participants request this assistance, an assessment of the baby's sleep and related factors is completed by the research nurse and this assessment is then brought to a meeting of the Sleep team and appropriate intervention discussed. The research nurse then takes a suggested plan back to the family and works alongside the family to assist implementing the agreed approach. Support by way of phone calls or visits will be given as required. The interventions used are matched to each family with the following modified "extinction" techniques being preferred: Parental Presence, Controlled Comforting and 'Camping Out' [66,82].

##### COMBO group

Those in the Combo group receive all FAB and SLEEP interventions as described above. Some are combined so that there are 9 intervention visits in total.

### Outcome measures

The timing of all outcome measures is presented in Table 1. The primary outcome measures are weight velocity at 0-6, 6-12, and 12-24 months; and body mass index z score at 24 months of age. All other measures are for secondary outcomes.

**Table 1:** Outcome measures for the study

		Time point							
	**Preg**	**Birth**	**3 w**	**7 w**	**4 m**	**6 m**	**12 m**	**18 m**	**24 m**

Cord blood		x							

Anthropometry - child^a^						x	x	x	x

Anthropometry - parent^b^						x	x	x	x

Sleep (child)									
Questionnaire/diary			x		x	x	x	x	x
Accelerometry						x	x		x

Sleep (parents)					x	x	x	x	x

Breast feeding and introduction of solids			Brief monthly phone call from 7 to 23 weeks						

Diet in child							x		x

Feeding behaviours								x	

Physical activity (child)									
Questionnaire					x	x	x	x	x
Accelerometry					x	x	x	x	x
Sedentary behaviour						x	x		x
Physical activity (parent)						x	x		x

Maternal depression	x				x		x		x

Paternal depression	x						x		x

Infant Temperament						x			x

Parenting									
skills & efficacy		x			x	x	x		x
support						x			x

Family quality of life							x		x

See text for specific methods

^a^Weight, length, and head and waist circumferences at each time point

^b^Weight, height and waist circumference of mother and father at 6 and 24 months, weight and waist circumference in mother at 12 months, and weight in mother at 18 months

#### Anthropometry

Trained measurers blinded to group allocation will conduct all outcome measurements following standard protocols, in general as used by the WHO studies that established the WHO ideal growth charts [83]. As waist circumference is not specified in the WHO protocols, waist circumference for infants up to 2 years of age will be measured just above the plane connecting the superior iliac crests, with the infant lying down. In parents, the natural waist (the narrowest part of the torso) will be measured. Pre-pregnancy maternal weight will be self-reported. All other weights will be measured in standard clothing conditions (singlet and no nappy for babies, singlet and/or underwear for older children) using regularly calibrated electronic scales (Tanita WB-100 MA/WB -110 MA). Length will be measured using a Rollameter 100c length board (Harlow Healthcare, UK), head circumference using a Seca 212 measuring tape (Seca, Germany) and abdominal circumferences using a Rosscraft Anthropometric tape (Rosscraft Innovations Inc, USA). All heights will be measured using a Harpenden stadiometer (Holtain Ltd, UK) in the clinic setting or a Leicester Height Measure (Harlow Healthcare, UK) when parents request a home visit for measuring. All measurements will be conducted in sequence as follows (mother (M), father (F), baby (B), weight (w), height (h), length (l), waist circumference (wc), head circumference(hc)): Mh, Mwc, Mh, Mwc, Bhc, Mw, Bw, Mw, Bw, Bwc, Bl, Bwc, Bl, Bhc, Fw, Fh, Fwc, Fw, Fh, Fwc.

A third measurement will be made if the duplicate measures indicated above are not within 0.1 kg for weight, 0.5 cm for height, 0.7 cm for length, 1.0 cm for waist circumference, and 0.5 cm for Head circumference. Table 1 details the measurements at each time point.

#### Breastfeeding

To gain a more accurate measure of breastfeeding status and when solids are introduced, participants will be interviewed by brief phone call every 4 weeks when their infant is aged 3-27 weeks, and asked to provide information on changes in breastfeeding, or infant formula feeding, to the nearest week. Qualitative interviews will also be conducted with a subset of women to explore the role of perceived insufficient milk supply in shortening the duration of breastfeeding, and to describe the experiences of women who exclusively breastfeed successfully to 6 months.

#### Dietary intake and habits

Dietary intake will be assessed by a validated food-frequency questionnaire (FFQ) at 12 and 24 months. This consists of 87 foods or food groupings and assesses frequency of intake across seven categories ranging from less than once per month to number of times per day. Serving size is measured using a combination of child palm size and usual counts (eg. 1 slice of bread). The assessment of leftovers is also incorporated into each question. Maternal feeding practices will be assessed using the Comprehensive Feeding Practices Questionnaire [84] at 18 months and by the Caregiver's Feeding Styles Questionnaire at 24 months [85]. Food security was measured at baseline using questions derived from the New Zealand National Nutrition Survey1997 [2].

#### Physical activity and inactivity

The primary measure of physical activity will be obtained from Actical accelerometers (Mini-Mitter, Bend, OR) attached by specially made belts to the child's ankle (6 months) or waist (12 and 24 months) and worn over 5 consecutive 24-hr periods. Given the lack of validated cut-offs for determining intensity of activity in infancy, activity accelerometry will be measured as counts/minute. Questionnaires will assess the amount of active outdoor and indoor play, stroller and car seat use, and television viewing; and family rules around television use. Parental physical activity will be assessed using the short form of the New Zealand Physical Activity Questionnaire [86,87]. Motor skills will be assessed by using age appropriate questions from WHO developmental questions [88] as well as the "ages and stages" questionnaire [89].

#### Sleep

The quantity and quality of sleep will be assessed by questionnaire and 2-day sleep diaries (5-minute blocks) at multiple time points (see Table 1). The questionnaires were developed from the consensus opinion of the researchers and using items from a range of validated sleep questionnaires [90,91] and national surveys. The format has been standardised to ask for usual behaviour over the last 2 weeks, in particular time put to bed, time taken to settle to sleep, number and usual duration of awakenings and wake up time. We asked parents to answer similar questions about their own sleep and also to provide a self-rating of adequacy of duration and quality of sleep. Specific questions around pre-sleep routines and sleep environment has also been included. Parental fatigue levels will be assessed using the brief fatigue assessment scale [92].

Infant sleep will also be objectively measured using Actical accelerometers (Mini-Mitter, Bend, OR) over 5 consecutive 24-hr periods as for physical activity assessment [93]. An algorithm developed specifically for the accelerometer will be used to identify infant sleep-wake states.

#### Biological samples

Cord blood will be collected and stored at the time of delivery. Genomic DNA will be prepared and the contribution of inherited genetic variants known to explain variation in weight tested for a role in weight gain in the children. Examples are the FTO and MC4R genes. The influence of environmental factors on genes will also be tested, including interactions and epigenetic changes.

#### Parenting and family life

Parenting skills and efficacy will be measured using four brief scales from the Longitudinal Study of Australian Children (LSAC) measuring warmth, irritability, consistency and overprotection (24 items extracted from Wave 1 interview schedule 1 and parent questionnaire 1) [94]. Maternal attachment to infant, and infant adaptability will be assessed by two subscales from the Parent Stress Index [95], while usual discipline practices will be assessed using an age appropriate list of positive and negative behaviours from 6 months of age. Family chaos will be assessed [96] at 18 months as will family quality of life [97] at 12 and 24 months.

#### Parental mental state, stress and support

The Edinburgh Postnatal Depression Scale will be used for both parents at baseline, 4 and 12 months [98,99]. The Depression Anxiety and Stress scale will be used with both parents at 2 years [100].

#### Infant temperament

The 30 item Colorado Childhood Temperament Inventory [101] will be used at 6 months and 2 years of age, partly because it contains a subscale that looks at reaction to food.

### Data analysis

Analyses will be conducted using the intention to treat principle (retrospective exclusions will be made where participants are subsequently found to have not satisfied inclusion/exclusion criteria) although some missing data is anticipated due to dropout. Linear mixed models will be used to compare arms of the trial for continuous outcomes (for example weight or BMI z-score) using an appropriate covariance structure for the repeated measures (selected using Akaike's and Bayesian Information Criteria). Binary and ordinal logistic mixed models will be used where the outcome is categorical (for example, moderate or major sleep problem at 2 years). Models will control for known predictors of the outcome of interest (including parity and birth weight). The linearity of associations for continuous predictors will be examined using fractional polynomials, with transformations used when necessary. Appropriate residual plots will be examined to identify problems with model assumptions. Missing data are anticipated to be either missing completely at random or missing at random, in which case maximum likelihood estimates will be unbiased, but sensitivity analyses will be performed to examine possible effects on conclusions should this assumption not hold. In all cases, 95% confidence intervals will be reported. Statistical significance will be determined by *p *< 0.05 and all tests will be two-sided.

## Discussion

This study looks directly at modifying growth in early infancy--a time when it is likely to be easiest to set up patterns of behaviour around food, activity and sleep that appear to be associated with healthier child and adult weight. Three similar studies are currently being conducted in Australia which all focus on first time mothers [102-104]. The 3 Australian studies and this New Zealand study will prospectively collect an identical minimum dataset, and have agreed to, after individual analysis of each study, combine our data in a prospective meta-analysis [105]. It should be noted that this New Zealand study is the only one to include multi-parous mothers as well as a specific intervention focused on sleep. As such, it is one of only a few studies deliberately using sleep as an intervention to prevent or manage overweight status [69,106,107]. As there is considerable controversy around using behavioural methods to alter infant sleep patterns, this study is taking considerable effort to look at any effect of these interventions on parenting and maternal attachment, as well as infant behaviour. If these interventions prove to be successful, the primary prevention of obesity in infancy could have a major impact on children over their life course, contributing to lower cardiovascular risk, hypertension, obstructive sleep apnoea, type 2 diabetes, and joint and bone problems.

## Competing interests

The authors declare that they have no competing interests.

## Authors' contributions

Professor BT and Associate Professor RT are co-principal investigators and prepared the first draft of this paper. The other authors have had significant input into the design and development of the study, and have commented on and edited this manuscript. All authors read and approved the final manuscript.

## Pre-publication history

The pre-publication history for this paper can be accessed here:

http://www.biomedcentral.com/1471-2458/11/942/prepub
